# Enhancement of methanogenesis by electric syntrophy with biogenic iron‐sulfide minerals

**DOI:** 10.1002/mbo3.647

**Published:** 2018-06-06

**Authors:** Souichiro Kato, Kensuke Igarashi

**Affiliations:** ^1^ Bioproduction Research Institute National Institute of Advanced Industrial Science and Technology (AIST) Sapporo Japan; ^2^ Division of Applied Bioscience Graduate School of Agriculture Hokkaido University Sapporo Japan

**Keywords:** electric syntrophy, *Geobacter*, iron sulfide, Methanogenesis, *Methanosarcina*

## Abstract

Recent studies have shown that interspecies electron transfer between chemoheterotrophic bacteria and methanogenic archaea can be mediated by electric currents flowing through conductive iron oxides, a process termed electric syntrophy. In this study, we conducted enrichment experiments with methanogenic microbial communities from rice paddy soil in the presence of ferrihydrite and/or sulfate to determine whether electric syntrophy could be enabled by biogenic iron sulfides. Although supplementation with either ferrihydrite or sulfate alone suppressed methanogenesis, supplementation with both ferrihydrite and sulfate enhanced methanogenesis. In the presence of sulfate, ferrihydrite was transformed into black precipitates consisting mainly of poorly crystalline iron sulfides. Microbial community analysis revealed that a methanogenic archaeon and iron‐ and sulfate‐reducing bacteria (*Methanosarcina*,* Geobacter*, and *Desulfotomaculum*, respectively) predominated in the enrichment culture supplemented with both ferrihydrite and sulfate. Addition of an inhibitor specific for methanogenic archaea decreased the abundance of *Geobacter*, but not *Desulfotomaculum*, indicating that *Geobacter* acquired energy via syntrophic interaction with methanogenic archaea. Although electron acceptor compounds such as sulfate and iron oxides have been thought to suppress methanogenesis, this study revealed that coexistence of sulfate and iron oxide can promote methanogenesis by biomineralization of (semi)conductive iron sulfides that enable methanogenesis via electric syntrophy.

## 1 INTRODUCTION

In anaerobic environments, where available energy sources are often restricted, a symbiotic relationship based on energy and electron transfer between multiple microbial species, specifically termed syntrophy, is a very important metabolic process. In particular, the syntrophic interaction between chemoheterotrophic bacteria and methanogenic archaea contributes greatly to CH_4_ production in diverse anaerobic environments and has a great influence on the global carbon cycle (Stams, 1994; Thauer, Kaster, Seedorf, Buckel, & Hedderich, 2008). In the syntrophic methanogenesis reaction, interspecies electron transfer (IET) from chemoheterotrophic bacteria to methanogenic archaea had been considered to occur only via diffusion of small molecules (e.g., H_2_ and formate) as electron carriers (Kato & Watanabe, 2010; Schink, 1997). Recent studies, however, have disclosed that IET can be mediated by electric currents flowing through conductive solid materials, a process that is specifically termed electric syntrophy or direct IET (Kato, 2015; Kouzuma, Kato, & Watanabe, 2015; Malvankar & Lovley, 2014). In addition to naturally occurring iron oxide minerals such as magnetite (Cruz Viggi et al., 2014; Kato, Hashimoto, & Watanabe, 2012a,2012b; Li et al., 2015; Liu et al., 2015; Yamada, Kato, Ueno, Ishii, & Igarashi, 2015; Zhuang, Tang, Wang, Hu, & Zhou, 2015), biological apparatus such as pili (Rotaru et al., 2014; Shrestha et al., 2013; Summers et al., 2010) and artificial conductive materials such as graphite (Chen et al., 2014), activated carbon (Liu et al., 2012; Rotaru et al., 2014), and carbon nanomaterials (Lin et al., 2017) have been reported as conduits of the electric currents that mediate syntrophy (reviewed in Cheng & Call, 2016; Lovley, 2017; Barua & Dhar, 2017). Because these studies have also pointed out that methanogenesis based on electric syntrophy is more efficient than methanogenesis based on diffusive transport of small molecules, supplementation with conductive particles has been proposed as a novel biotechnology for improvement of anaerobic wastewater treatment systems (Baek, Kim, & Lee, 2016; Dang et al., 2016; Sasaki et al., 2018; Zhao et al., 2016).

Most iron exists as divalent or trivalent minerals in natural environments. In anaerobic environments where methanogenesis occurs, divalent minerals such as iron sulfides predominate (Nielsen, Risgaard‐Petersen, Fossing, Christensen, & Sayama, 2010; Weber, Achenbach, & Coates, 2006). In such environments, iron sulfides are continuously formed via reduction in iron and sulfur compounds by dissimilatory iron‐reducing bacteria (DIRBs) and sulfate‐reducing bacteria (SRBs), respectively (Coleman, Hedrick, Lovley, White, & Pye, 1993; Flynn, O'Loughlin, Mishra, DiChristina, & Kemner, 2014; Gramp, Bigham, Jones, & Tuovinen, 2010; Igarashi & Kuwabara, 2014; Igarashi, Yamamura, & Kuwabara, 2016). It has been reported that iron sulfide minerals found in natural environments could have conductive (or semiconductive) properties (Malvankar, King, & Lovley, 2015; Nakamura et al., 2010; Yamamoto et al., 2017). Furthermore, it has been reported that biogenic iron sulfide minerals such as mackinawite produced by DIRB/SRB (e.g., *Shewanella* spp. and *Desulfopila* spp.) function as electrical conduits for long‐distance electron transfer (Enning et al., 2012; Kondo, Okamoto, Hashimoto, & Nakamura, 2015; Nakamura et al., 2010), as in the case of conductive iron oxide minerals (Kato, Hashimoto, & Watanabe, 2013; Kato, Kai, Nakamura, Watanabe, & Hashimoto, 2010; Nakamura, Kai, Okamoto, Newton, & Hashimoto, 2009). From these findings, we can assume that iron sulfide minerals may also mediate methanogenesis via electric syntrophy. However, electric syntrophy reactions dependent on (semi)conductive iron sulfides have not yet been experimentally demonstrated.

In this study, methanogenic microbial communities were enriched in the presence of sulfate and/or nonconductive, amorphous iron oxides (ferrihydrite). We then tested the hypothesis that biogenic iron sulfides enabled methanogenesis by mediating electric syntrophy. The experiments involved measurements of methanogenic activity, determination of mineral species formed, and investigation of microbial community structure in each enrichment culture.

## 2 EXPERIMENTAL PROCEDURES

### 2.1 Enrichment cultures

Methanogenic microbial communities were enriched in vials (68 ml in capacity) filled with 20 ml of an inorganic medium (PSN medium) supplemented with 20 mmol/L sodium acetate as described previously (Kato et al., 2012b). Fifty milligrams (wet weight) of rice paddy field soil was inoculated as a source of microorganisms. Ferrihydrite was prepared as described elsewhere (Lovley & Phillips, 1986) and was used to supplement +Fer and +Fer/${\text{SO}}_{4}^{2-}$ cultures to give a final Fe concentration of 20 mmol/L. Sodium sulfate (final concentration, 10 mmol/L) was used to supplement +SO_4_ and +Fer/${\text{SO}}_{4}^{2-}$ cultures. For the cultures of methanogenesis inhibition, bromoethane sulfonate (BES) (final concentration, 5 mmol/L) was added as a specific inhibitor of methanogenic archaea to the same medium used for the enrichment cultures. All cultures were incubated at 30°C without shaking under an atmosphere of N_2_/CO_2_ (80/20). CH_4_ and H_2_ in the gas phases were measured, using a gas chromatograph (GC‐2014, Shimadzu, Kyoto, Japan) as described previously (Kato, Sasaki, Watanabe, Yumoto, & Kamagata, 2014). At the early stationary phases, iron minerals were collected by centrifugation and were dried under N_2_ atmosphere in an anaerobic chamber (Vinyl Anaerobic Chambers Type C, Coy Laboratory Products, Grass Lake, MI). The X‐ray diffraction (XRD) and the X‐ray fluorescence (XRF) spectra of the iron minerals were obtained, using an X‐ray diffractometer (Ultima X, Rigaku, Tokyo, Japan) and X‐ray fluorescence spectrometer (SEA5120A, Hitachi High‐Technologies, Tokyo, Japan), respectively. The culture experiments were conducted in triplicate, and Student's *t*‐test was used to determine the significance of treatment effects.

### 2.2 Microbial community analysis

After five successive subcultures, enriched microorganisms were collected by centrifugation. DNA was extracted using the FAST DNA Spin Kit for soil (MP Biomedicals, Irvine, US) according to the manufacturer's instructions. Partial 16S rRNA gene fragments were amplified by PCR with a primer pair of 27F and 533R for bacteria and A25F and A958R for archaea, as described previously (Kato et al., 2010, 2015). PCR products were purified using a QIAquick PCR Purification Kit (QIAGEN, Hilden, Germany), ligated into pGEM‐T Easy Vector (Promega, Madison, US) and cloned into *E. coli* JM109 competent cells (Promega). Sequences of the cloned PCR products were determined at the Biomedical Center, TAKARA Bio (Kusatsu, Japan). The nucleotide sequence data reported here have been submitted to GenBank under Accession Nos. LC111538–LC111564.

### 2.3 Quantitative PCR

Quantitative real‐time PCR was performed using a LightCycler 96 real‐time PCR system (Roche, Basel, Switzerland) and THUNDERBIRD SYBR qPCR Mix (Toyobo, Osaka, Japan) according to the manufacturer's instructions. Table S3 lists the primers used for the qPCR analyses. After an initial denaturation at 95°C for 1 min, targets were amplified by 40 cycles of denaturation for 15 s at 95°C followed by annealing and extension for 45 s at 60°C. Fluorescence was measured at the end of the extension step. The PCR amplicons were assessed via a melting‐curve analysis to check for successful amplification. At least two separate trials were conducted for each DNA sample. Standard curves were generated with serially diluted PCR products amplified using the respective primer sets.

## 3 RESULTS AND DISCUSSION

### 3.1 Enrichments of methanogenic microbial communities with ferrihydrite and/or sulfate

**Figure 1:**
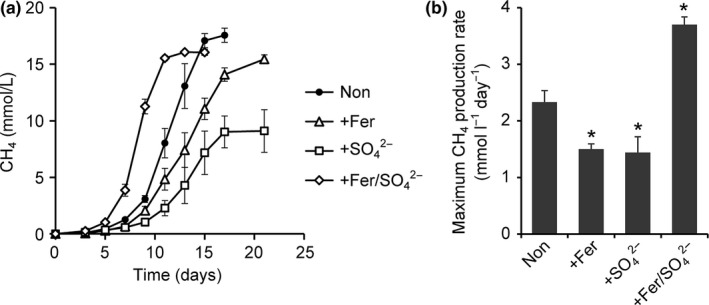
Effects of supplementation with sulfate and/or iron oxides on methanogenic activities of cultures enriched with 20 mmol/L acetate as a substrate. (a) Time‐courses of CH_4_ production in the enrichments supplemented with no additives (Non), ferrihydrite (+Fer), sulfate (+${\text{SO}}_{4}^{2-}$), and both ferrihydrite and sulfate (+Fer/${\text{SO}}_{4}^{2-}$). (b) The maximum CH_4_ production rates calculated from the data in (a). Asterisks represent a significant difference (*p* < .05) from the Non enrichment. Data are presented as the means of three independent cultures, and error bars represent standard deviations

Enrichment culture experiments were conducted to determine whether or not electric syntrophy could be mediated by biogenic iron sulfide minerals derived from simultaneous reduction in Fe(III) and sulfate. Cultures of methanogenic microbial communities from rice paddy field soil enriched with 20 mmol/L acetate as the sole energy and carbon sources and supplemented with both ferrihydrite (20 mmol/L as Fe) and sulfate (10 mmol/L) are hereafter referred to as the +Fer/${\text{SO}}_{4}^{2-}$ enrichments. Enrichment cultures with no supplements or supplemented with either ferrihydrite or sulfate, but not both, were also conducted as controls. They are hereafter referred to as the Non, +Fer, and +${\text{SO}}_{4}^{2-}$ enrichments, respectively. After five successive subcultures, the rate of CH_4_ production by each culture was measured (Figure 1). In the Non enrichment, approximately 17.6 mmol/L of CH_4_ was produced from 20 mmol/L acetate (Figure 1a), suggesting that methanogenesis was the major electron sink in the enrichment culture. Rates of CH_4_ production in the +Fer and +${\text{SO}}_{4}^{2-}$ enrichments were significantly suppressed compared to the Non enrichment (Figure 1b). This observation is consistent with previous reports; the explanation is that DIRBs and SRBs outcompete methanogenic archaea for common substrates (e.g., acetate) (Achtnich, Bak, & Conrad, 1995; Chidthaisong & Conrad, 2000; Kato et al., 2012b; Lueders & Friedrich, 2002; Yamada, Kato, Ueno, Ishii, & Igarashi, 2014). In contrast, a significant enhancement of methanogenesis was observed in the +Fer/${\text{SO}}_{4}^{2-}$ enrichment. These results clearly demonstrated that the presence of ferrihydrite and sulfate together enhanced methanogenesis, whereas the presence of either substrate alone did not. This result supports the hypothesis that electric syntrophy was mediated by biogenic iron sulfide minerals.

### 3.2 Analyses of the biomineralization products

**Figure 2:**
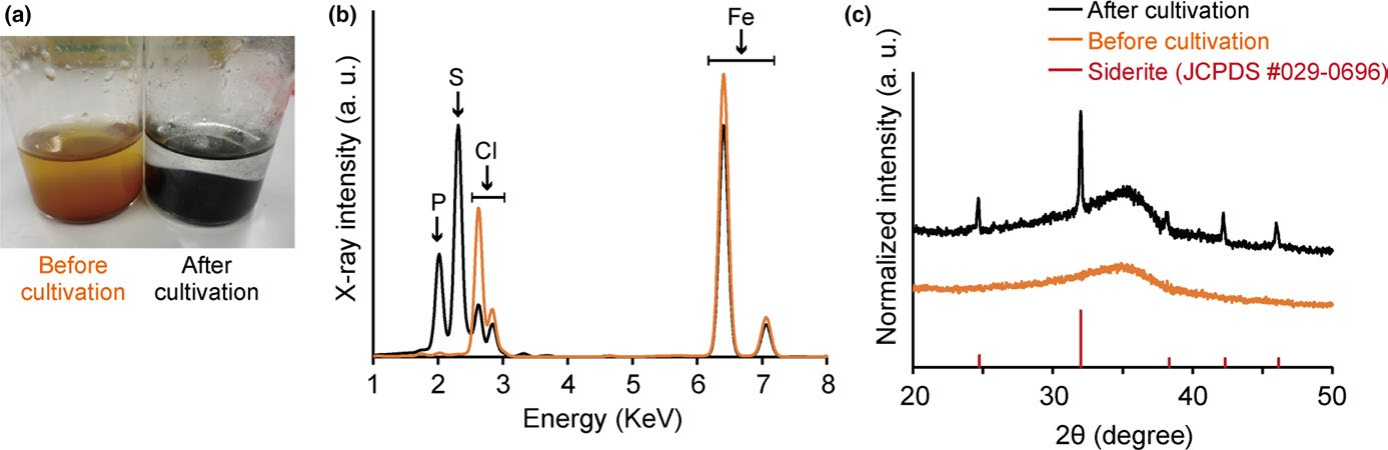
Biomineralization of iron sulfides in the enrichment culture supplemented with both ferrihydrite and sulfate. (a) Changes in color of minerals before and after cultivation. The X‐ray fluorescence (XRF) spectra (b) and the powder X‐ray diffraction (XRD) patterns (c) of the minerals taken before (orange line) and after (black line) cultivation. In (c), the reflection pattern of pure siderite (FeCO_3_) is shown under the sample patterns

During the +Fer/${\text{SO}}_{4}^{2-}$ enrichment, ferrihydrite particles that were reddish brown in color turned into black precipitates (Figure 2a). The precipitates were collected from the +Fer/${\text{SO}}_{4}^{2-}$ enrichment before and after cultivation, dried under anoxic conditions, and subjected to the XRF and the XRD analyses to identify the Fe species contained in the black precipitates. The XRF analysis showed that the black precipitates generated during the +Fer/${\text{SO}}_{4}^{2-}$ enrichment contained more phosphorous and much more sulfur than the minerals recovered from samples before cultivation (Figure 2b), indicating that the black precipitates contained iron sulfide and minor amounts of phosphate minerals. The XRD analysis of the red–brown precipitate before cultivation showed only a broad peak characteristic of ferrihydrite (Figure 2b). In contrast, the black precipitate after cultivation showed, in addition to the broad ferrihydrite peak, several sharp peaks, an indication of significant alteration of the mineral phases. These peaks corresponded to siderite (FeCO_3_), an indication of microbial reduction in ferrihydrite into ferrous iron during cultivation. Although XRF analysis indicated the presence of iron sulfide in the black precipitate, XRD analysis failed to identify any peaks that corresponded to crystalline iron sulfide minerals. It is therefore likely that the iron sulfide in the black precipitates was amorphous or poorly crystalline species (Jeong, Lee, & Hayes, 2008). These results suggest that the black precipitate was a mixture of various mineral species, including amorphous iron sulfide, ferrihydrite, iron phosphate, and siderite. Among these mineral species, ferrihydrite, iron phosphate, and siderite are electrical insulators. On the other hand, it has been reported that some iron sulfide species have electric conductivity (Enning et al., 2012; Kondo et al., 2015; Nakamura et al., 2010). Especially, Enning et al. (2012) demonstrated electrical conductivity of biomineralization products generated by iron‐corrosive SRBs mainly consisting of amorphous iron sulfide. Collectively, it is supposed that amorphous iron sulfide confer electrical conductivity to the black precipitate in the +Fer/${\text{SO}}_{4}^{2-}$ enrichment and mediate electric syntrophy.

### 3.3 Dominant microorganisms in the enrichment cultures

Clone library analysis of both the archaeal and bacterial 16S rRNA genes was conducted to determine the dominant microorganisms in the enrichment cultures. A phylotype was defined as a unique clone or a group of clones with sequence similarity >98%. All phylotypes obtained in this study are summarized in Tables S1 and S2. Only one archaeal phylotype (FeAcA001, 99% identity to *Methanosarcina barkeri*) was recovered from all the enrichment cultures (Table S1), and no other archaeal phylotype was recovered from any of the enrichment cultures. These results suggest that *Methanosarcina* spp. generated CH_4_ in all the enrichment cultures.

**Figure 3:**
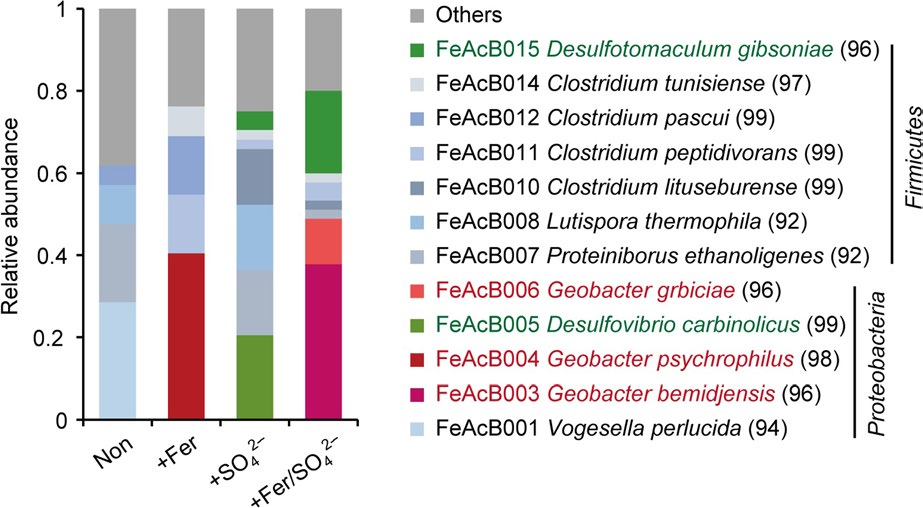
Phylogenetic distribution of bacterial 16S rRNA gene clones recovered from the enrichment cultures supplemented with no additives (Non), ferrihydrite (+Fer), sulfate (+${\text{SO}}_{4}^{2-}$), and both ferrihydrite and sulfate (+Fer/${\text{SO}}_{4}^{2-}$). The dominant phylotypes (>10% in at least one enrichment) and their closest relatives (sequence identity, %) are shown in the legends. The phylotypes closely related to Fe(III)‐reducing and sulfate‐reducing bacteria are highlighted in red and green, respectively

A total of 26 phylotypes were obtained from the clone library analysis targeting bacteria (Table S2). Figure 3 summarizes the relative abundances of the dominant phylotypes. The dominant bacteria in the cultures supplemented with ferrihydrite (i.e., the +Fer and +Fer/${\text{SO}}_{4}^{2-}$ enrichments) were *Geobacter* spp., known as DIRBs (Figure 3, highlighted in red color). However, the *Geobacter* spp. that predominated in the +Fer and +Fer/${\text{SO}}_{4}^{2-}$ enrichments were different at the phylotype level; the dominant *Geobacter* in the +Fer enrichment was phylotype FeAcB004 (98% identity to *Geobacter psychrophilus*), whereas the dominant *Geobacter* in the +Fer/${\text{SO}}_{4}^{2-}$ enrichment were FeAcB003 (96% identity to *Geobacter bemidjensis*) and FeAcB006 (96% identity to *Geobacter grbiciae*). Because there have been no reports indicating that *Geobacter* spp. are capable of utilizing sulfate as an electron acceptor, it is unlikely that the observed difference in the dominant *Geobacter* types was simply due to the presence or absence of sulfate. Because previous reports have indicated that some *Geobacter* spp. are involved in electric syntrophy‐facilitated methanogenesis (Kato et al., 2012b; Morita et al., 2011; Rotaru et al., 2014, 2014), it is probable that the *Geobacter* spp. that dominated in the +Fer/${\text{SO}}_{4}^{2-}$ enrichments (FeAcB003 and FeAcB006) contributed to syntrophic methanogenesis in addition to simple Fe(III) reduction.

Dominance of SRBs was apparent in the cultures supplemented with sulfate (i.e., the +${\text{SO}}_{4}^{2-}$ and +Fer/${\text{SO}}_{4}^{2-}$ enrichments) (Figure 3, highlighted in green color). As was the case for *Geobacter* spp. in the ferrihydrite‐supplemented treatments, the dominant SRB types differed between the +${\text{SO}}_{4}^{2-}$ and +Fer/${\text{SO}}_{4}^{2-}$ enrichments: phylotype FeAcB005 (99% identity to *Desulfovibrio carbinolicus*) in the +SO_4_ enrichment and FeAcB015 (96% identity to *Desulfotomaculum gibsoniae*) in the +Fer/${\text{SO}}_{4}^{2-}$ enrichment. There is no clear explanation for this observed difference in dominant SRB types. Because some SRB strains, including *Desulfotomaculum* spp., have been reported capable of reducing Fe(III) (Junier et al., 2009; Lovley, 1993), a difference in the availability of Fe(III) might have caused the changes in the SRB communities. Other plausible factors include a change in redox potential due to the presence of ferrihydrite, toxicity of ferrous ions, and a decrease in availability of acetate due to coexistence of *Geobacter* spp.

### 3.4 The effects of a methanogenic inhibitor on DIRBs and SRBs

**Figure 4:**
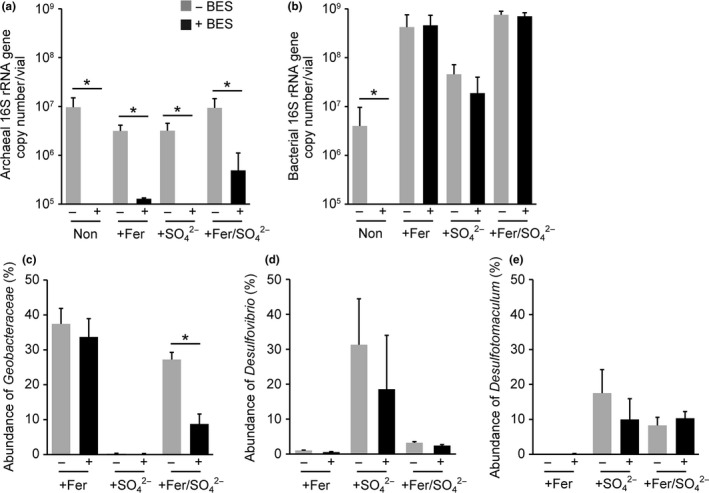
The abundances of archaea (a), bacteria (b), Geobacteraceae (c), *Desulfovibrio* (d), and *Desulfotomaculum* (e) in the enrichment cultures assessed via the qPCR method. (c–e) The relative abundances (%) were calculated as a relative ratio to the sum of total bacteria and total archaea. Asterisks represent a significant difference (*p* < .05) between results in the presence or absence of a methanogenesis inhibitor (5 mmol/L BES). Data are presented as the means of three independent cultures, and error bars represent standard deviations

In electric syntrophy‐facilitated methanogenesis, chemoheterotrophic bacteria and methanogenic archaea are in an interdependent relationship. The growth of chemoheterotrophic bacteria, which accompanies electric syntrophy, was therefore indirectly suppressed by a specific inhibitor of methanogenesis (Kato et al., 2012b). To identify microorganisms involved in electric syntrophy, each enrichment was further cultivated in the presence or absence of BES, a specific inhibitor of methanogens, and the abundances of total bacteria, archaea, and dominant DIRB and SRB species were measured via the quantitative PCR (qPCR) method (Figure 4).

Methane production was completely suppressed in the cultures supplemented with 5 mmol/L of BES (data not shown). Supplementation of BES reduced the abundance of total archaea to less than 5% of those in the BES‐free cultures (Figure 4a), suggesting that the growth of archaea was completely suppressed by BES, considering the inoculation size (5% v/v). In the absence of BES, the total bacterial abundances were significantly higher in the enrichments supplemented with ferrihydrite and/or sulfate than in the Non enrichment (Figure 4b). A significant decrease caused by BES supplementation was observed only in the Non enrichment; no significant effect was observed in other enrichments (Figure 4b). These results suggest that only archaea contributed to the decomposition of acetate in the Non enrichment, whereas both archaea and bacteria contributed under other conditions.

The abundance of *Geobacter* spp. (Figure 4c) was high under ferrihydrite‐supplemented conditions (the +Fer and +Fer/${\text{SO}}_{4}^{2-}$ enrichments), a result consistent with the clone library analysis. The abundance of *Geobacter* spp. was not significantly affected by supplementation with BES in the +Fer enrichment, suggesting that *Geobacter* spp. acquired the energy required for growth via only Fe(III) reduction. By contrast, supplementation with BES significantly lowered the abundance of *Geobacter* spp. in the +Fer/${\text{SO}}_{4}^{2-}$ enrichment. Under the BES‐supplemented conditions, *Geobacter* spp. can acquire energy only via Fe(III) reduction. The only possible explanation of the decrease in the abundance of *Geobacter* spp. by BES supplementation is that *Geobacter* spp. performed energy metabolisms dependent on the presence of methanogens, that is, electric syntrophy, in addition to Fe(III) reduction.

The results of qPCR analyses of the two types of SRBs were consistent with the clone library analyses; *Desulfovibrio* spp. were more abundant in the +${\text{SO}}_{4}^{2-}$ enrichment, whereas *Desulfotomaculum* spp. were the major SRBs in the +Fer/${\text{SO}}_{4}^{2-}$ enrichment (Figure 4d and e). Supplementation with BES did not significantly alter the abundance of *Desulfovibrio* and *Desulfotomaculum*, suggesting that these SRBs do not perform syntrophic metabolisms with methanogenic archaea; instead, they simply acquire energy through sulfate reduction.

### 3.5 Estimation of metabolic and electron flow in each enrichment culture

**Figure 5:**
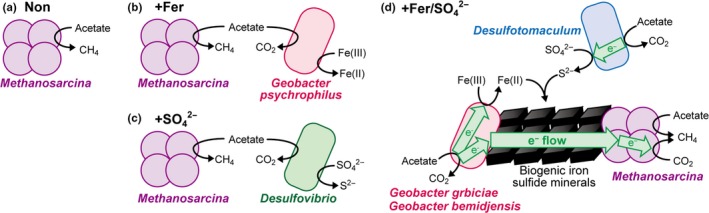
Schematic illustration of the putative metabolic reactions, electron flows, and microbial species involved in each enrichment culture

Based on the results obtained in this study, we estimated the metabolic reactions and electron flows occurring in each enrichment culture and the microbial species involved (Figure 5). In the Non enrichment, an aceticlastic methanogen, *Methanosarcina* spp., was thought to simply convert acetate to CH_4_ (Figure 5a). When ferrihydrite was supplemented (Figure 5b), the number of bacteria increased greatly, and *Geobacter* spp. became dominant (Figures 3 and 4). *Geobacter* spp. are thought to compete with methanogenic archaea for acetate, and such competition would result in a decrease in the rate of CH_4_ production (Figure 1). Supplementation with sulfate (Figure 5c) led to a dominance of SRBs, especially *Desulfovibrio* spp. (Figures 3 and 4). Similar to the results of the +Fer enrichment, competition between SRBs and methanogens could have caused the rate of CH_4_ production to decrease (Figure 1).

In contrast, the metabolic flow and the microbial species involved dramatically changed when the medium was simultaneously supplemented with ferrihydrite and sulfate. Unlike the case of the +Fer and +${\text{SO}}_{4}^{2-}$ enrichments, CH_4_ production was significantly enhanced in the +Fer/${\text{SO}}_{4}^{2-}$ enrichment (Figure 1). Blackish minerals, mainly consisting of iron sulfides, were generated in the +Fer/${\text{SO}}_{4}^{2-}$ enrichment culture (Figure 2). Microorganisms classified as both DIRB and SRB were predominant in the +Fer/${\text{SO}}_{4}^{2-}$ enrichment (Figure 3). Notably, the dominant DIRB and SRB species in the +Fer/${\text{SO}}_{4}^{2-}$ enrichment differed from those in the +Fer and +${\text{SO}}_{4}^{2-}$ enrichments, respectively. Results of additional cultivation experiments with supplemental BES suggested that *Geobacter* spp. performed syntrophic metabolisms with methanogenic archaea (Figure 4c). Based on these observations, we concluded that the following metabolisms and electron flows would occur in the +Fer/${\text{SO}}_{4}^{2-}$ enrichment (Figure 5d). Biomineralization of iron sulfides occurred via generation of ferrous iron and sulfides through reduction of ferrihydrite by *Geobacter* spp. (and possibly by *Desulfotomaculum* spp.) and reduction of sulfate by *Desulfotomaculum* spp., respectively. *Geobacter* spp. and *Methanosarcina* spp. then performed electric syntrophy with the biogenic iron sulfides as conduits to generate CH_4_. Methanogenesis based on electric syntrophy has been reported to be more efficient than the usual methanogenic processes (Cruz Viggi et al., 2014; Kato et al., 2012b; Li et al., 2015; Liu et al., 2015; Yamada et al., 2015; Zhuang et al., 2015), which could explain the increased rate of CH_4_ production in the +Fer/${\text{SO}}_{4}^{2-}$ enrichment.

### 3.6 Conclusion

This study demonstrated for the first time that biogenic iron sulfide minerals can enable methanogenesis based on electric syntrophy. It has been reported that the existence of electron acceptor compounds such as sulfate and ferrihydrite inhibit methanogenesis (Achtnich et al., 1995; Chidthaisong & Conrad, 2000; Kato et al., 2012b; Lueders & Friedrich, 2002; Yamada et al., 2014). The results of this study suggest that methanogenesis can be promoted when sulfate and ferrihydrite coexist. Because iron sulfide minerals are ubiquitous in anaerobic environments, it is highly possible that electric syntrophy with iron sulfides contributes greatly to methanogenesis and also to carbon cycles in natural environments. Further investigation on the effects of iron sulfide minerals in natural environments and also experiments using model co‐cultures of *Geobacter* spp. and *Methanosarcina* spp. with synthetic iron sulfide species will shed light on the importance of iron sulfide minerals for the global methane flux.

## CONFLICT OF INTEREST

The authors declare no conflict of interest.
