# Supplementary material for: Enhancement of methanogenesis by electric syntrophy with biogenic iron‐sulfide minerals
Source: Microbiologyopen. 2018 Jun 6;8(3):e00647. doi: 10.1002/mbo3.647 (PMC6436484; doi:10.1002/mbo3.647)
Supplement: Supplementary file 1 [file MBO3-8-e00647-s001.docx]

Table S1. The archaeal phylotypes detected in the enrichment cultures.

| Phylogenetic group | Phylotype | Closest relative (Similarity, %) | Number of clones | | | |
| --- | --- | --- | --- | --- | --- | --- |
|  |  |  | Non | +Fer | +SO_4_^2-^ | +Fer/SO_4_^2-^ |
| *Euryarchaeota* | FeAcA001 | AF028692 *Methanosarcina barkeri* strain Sar (99) | 12 | 15 | 14 | 15 |
|  |  | Total clone number | 12 | 15 | 14 | 15 |

Table S2. The bacterial phylotypes detected in the enrichment cultures.

| Phylogenetic group | Phylotype | Closest relative (Similarity, %) | Number of clones | | | |
| --- | --- | --- | --- | --- | --- | --- |
|  |  |  | Non | +Fer | +SO_4_^2-^ | +Fer/SO_4_^2-^ |
| *Betaproteobacteria* | FeAcB001 | KC706672 *Vogesella perlucida* strain C10-4m (94) | 12 |  |  |  |
|  | FeAcB002 | CP004143 *Pseudomonas denitrificans* ATCC 13867 (100) |  | 1 | 4 | 1 |
| *Deltaproteobacteria* | FeAcB003 | CP001124 *Geobacter bemidjiensis* strain Bem (96) |  |  |  | 17 |
|  | FeAcB004 | NR_043075 *Geobacter psychrophilus* strain P35 (98) |  | 17 |  |  |
|  | FeAcB005 | NR_074958 *Desulfovibrio magneticus* RS-1 (99) |  |  | 11 |  |
|  | FeAcB006 | NR_041826 *Geobacter grbiciae* strain TACP-5 (96) |  |  |  | 5 |
| *Firmicutes* | FeAcB007 | NR_044093 *Proteiniborus ethanoligenes* strain GW (96) | 8 |  | 7 | 1 |
|  | FeAcB008 | NR_041236 *Lutispora thermophila* strain EBR46 (92) | 4 |  | 7 |  |
|  | FeAcB009 | NR_043101 *Sporacetigenium mesophilum* strain ZLJ115 (99) | 1 | 4 |  | 4 |
|  | FeAcB010 | AY458860 *Clostridium lituseburense* strain EIB 6 (99) |  |  | 6 | 1 |
|  | FeAcB011 | NR_025019 *Clostridium peptidivorans* strain TMC4 (99) |  | 6 | 2 | 2 |
|  | FeAcB012 | NR_026322 *Clostridium pascui* strain DSM 10365 (99) | 2 | 6 |  |  |
|  | FeAcB013 | AJ012602 sulfate-reducing bacterium R-AcetonA70 (92) |  |  |  | 3 |
|  | FeAcB014 | AY187622 *Clostridium tunisiense* (97) |  | 3 | 1 | 1 |
|  | FeAcB015 | NR_037081 *Desulfotomaculum gibsoniae* DSM 7213 (96) |  |  | 2 | 9 |
|  | FeAcB016 | JQ897423 *Clostridium thiosulfatireducens* (99) | 2 | 2 |  |  |
|  | FeAcB017 | DQ117468 *Gracilibacter thermotolerans* strain JW/YJL-S1 (99) | 3 |  | 1 |  |
|  | FeAcB018 | JN650298 *Clostridium celerecrescens* strain SCTB133 (99) | 4 |  |  |  |
|  | FeAcB019 | AF427155 *Clostridium sp. LTR1* (98) |  | 2 |  |  |
|  | FeAcB020 | AB490809 *Christensenella minuta* (89) |  | 1 |  | 1 |
|  | FeAcB021 | GU129052 *Peptococcaceae* bacterium 34bG (85) | 1 |  | 1 |  |

Table S2. Continued.

| Phylogenetic group | Phylotype | Closest relative (Similarity, %) | Number of clones | | | |
| --- | --- | --- | --- | --- | --- | --- |
|  |  |  | Non | +Fer | +SO_4_^2-^ | +Fer/SO_4_^2-^ |
| *Firmicutes* | FeAcB022 | GU129927 *Clostridium magnum* strain FM5 (85) | 2 |  |  |  |
|  | FeAcB023 | AB627080 *Clostridium paraputrificum* strain: JCM 5237 (85) | 2 |  |  |  |
|  | FeAcB024 | NR_044972 *Sporobacter termitidis* strain SYR (92) |  |  | 1 |  |
|  | FeAcB025 | GQ461819 *Tissierella* sp. LBN 292 (99) |  |  | 1 |  |
|  | FeAcB026 | FR749956 *Tissierella creatinini* strain DSM 9508T (94) | 1 |  |  |  |
|  |  | Total clone number | 42 | 42 | 44 | 45 |

Table S3. PCR primers used in this study.

| Primer name | Sequences (5’ to 3’) | Target microorganisms | References |
| --- | --- | --- | --- |
| B1055F | ATGGYTGTCGTCAGCT | Bacteria | Harms *et al.,*2003 |
| B1392R | ACGGGCGGTGTGTAC | Bacteria | Harms *et al.,* 2003 |
| A349F | GYGCASCAGKCGMGAAW | Archaea | Takai and Horikoshi, 2000 |
| A958R | YCCGGCGTTGAMTCCAATT | Archaea | DeLong, 1992 |
| Geo494F | AGGAAGCACCGGCTAACTCC | Family *Geobacteraceae* | Holmes *et al*., 2002 |
| Geo825R | TACCCGCRACACCTAGT | Family *Geobacteraceae* | Holmes *et al*., 2002 |
| DEM116F | GTAACGCGTGGATAACCT | Genus *Desulfotomaculum* lineage I | Stubner, 2002 |
| DEM1164R | CCTTCCTCCGTTTTGTCA | Genus *Desulfotomaculum* lineage I | Stubner, 2002 |
| DSV691F | CCGTAGATATCTGGAGGAACATCMG | Genus *Desulfovibrio* | Fite *et al*., 2004 |
| DSV826R | ACAKCTAGCATCCATCGTTTACAGC | Genus *Desulfovibrio* | Fite *et al*., 2004 |

References:

Harms, G., Layton, A.C., Dionisi, H.M., Gregory, I.R., Garrett, V.M., Hawkins, S.A., Robinson, K.G., and Sayler, G.S. (2003) Real-time PCR quantification of nitrifying bacteria in a municipal wastewater treatment plant. *Environ Sci Technol* **37:** 343–351.

Takai, K., and Horikoshi, K. (2000) Rapid detection and quantification of members of the archaeal community by quantitative PCR using fluorogenic probes. *Appl Environ Microbiol* **66:** 5066–5072.

DeLong, E.F. (1992) Archaea in coastal marine sediments. *Proc Natl Acad Sci U S A* **89:** 5685–5689.

Holmes, D.E., Finneran, K.T., O’Neil, R.A., and Lovley, D.R. (2002) Enrichment of members of the family *Geobacteraceae* associated with stimulation of dissimilatory metal reduction in uranium-contaminated aquifer sediments. *Appl Environ Microbiol* **68:** 2300–2306.

Stubner, S. (2002) Enumeration of 16S rDNA of *Desulfotomaculum* lineage 1 in rice field soil by real-time PCR with SybrGreen detection. *J Microbiol Methods* **50:** 155–164.

Fite, A., Macfarlane, G.T., Cummings, J.H., Hopkins, M.J., Kong, S.C., Furrie, E., and Macfarlane, S. (2004) Identification and quantitation of mucosal and faecal desulfovibrios using real time polymerase chain reaction. *Gut* **53:** 523–529.
